# Predicting the risk of cognitive decline and speech impairment in the preclinical stage of Alzheimer's disease using structural MRI

**DOI:** 10.1002/alz.087212

**Published:** 2025-01-09

**Authors:** Qingyue Li, Alexandra König, Nicklas Linz, Fedor Levin, Martin Dyrba, Annika Spottke, Björn Falkenburger, Christoph Laske, Emrah Düzel, Frank Jessen, Inga Zerr, Jens Wiltfang, Josef Priller, Michael T. Heneka, Michael Wagner, Stefan Teipel, Stefanie Köhler

**Affiliations:** ^1^ Rostock University Medical Center, Rostock Germany; ^2^ Côte d'Azur University, Nice France; ^3^ ki:elements GmbH, Saarbrücken Germany; ^4^ German Center for Neurodegenerative Diseases (DZNE), Rostock Germany; ^5^ University of Bonn, Bonn Germany; ^6^ German Center for Neurodegenerative Diseases (DZNE), Bonn Germany; ^7^ German Center for Neurodegenerative Diseases (DZNE), Dresden Germany; ^8^ University Hospital Carl Gustav Carus, Technische Universität Dresden, Dresden Germany; ^9^ German Center for Neurodegenerative Diseases (DZNE), Tuebingen Germany; ^10^ Section for Dementia Research, Hertie Institute for Clinical Brain Research and Department of Psychiatry and Psychotherapy, University of Tuebingen, Tuebingen Germany; ^11^ University of Tuebingen, Tuebingen Germany; ^12^ Institute of Cognitive Neurology and Dementia Research (IKND), Otto‐von‐Guericke University, Magdeburg Germany; ^13^ German Center for Neurodegenerative Diseases (DZNE), Magdeburg Germany; ^14^ Excellence Cluster on Cellular Stress Responses in Aging‐Associated Diseases (CECAD), University of Cologne, Cologne Germany; ^15^ Faculty of Medicine and University Hospital Cologne, University of Cologne, Cologne Germany; ^16^ University Medical Center, Georg August University, Goettingen Germany; ^17^ German Center for Neurodegenerative Diseases (DZNE), Goettingen Germany; ^18^ Neurosciences and Signaling Group, Institute of Biomedicine (iBiMED), Department of Medical Sciences, University of Aveiro, Aveiro Portugal; ^19^ University Medical Center, University of Goettingen, Goettingen Germany; ^20^ Department of Psychiatry and Psychotherapy, Technical University of Munich, Munich Germany; ^21^ University of Edinburgh and UK DRI, Edinburgh UK; ^22^ Charité – Universitätsmedizin Berlin, Berlin Germany; ^23^ German Center for Neurodegenerative Diseases (DZNE), Berlin Germany; ^24^ University of Bonn Medical Center, Bonn Germany; ^25^ Luxembourg Centre for Systems Biomedicine (LCSB), University of Luxembourg, Luxembourg Luxembourg

## Abstract

**Background:**

Speech abnormalities are increasingly recognized as a manifestation of cognitive deficits in Alzheimer's disease (AD) and its preclinical and prodromal stages. Here, we investigated whether MRI measures of brain atrophy, specifically in the basal forebrain and cortical language areas, can predict cognitive decline and speech difficulties in older adults within the AD spectrum.

**Method:**

The ongoing Prospect‐AD study aims to develop an algorithm to automatically identify speech biomarkers in individuals with early signs of AD. We recruited participants for the Prospect‐AD study from the German national cohort studies DELCODE and DESCRIBE. Within Prospect‐AD, we captured speech recordings via automated phone calls in healthy controls and people with preclinical and prodromal AD. Speech features were extracted from the semantic verbal fluency (SVF) task of automated telephone conversations, using artificial intelligence‐based analysis of the audio recordings. We combined the speech data from Prospect‐AD with the clinical and imaging data from DELCODE and DESCRIBE, including socio‐demographic information, baseline AD diagnosis, neuropsychology, CSF amyloid status, and MRI scans. We used mixed‐effects regression to estimate the association of rates of cognitive decline and traits of automatically obtained speech features with regional atrophy.

**Result:**

To date, we enrolled 220 participants in the Prospect‐AD study, 103 of whom are included in this report. Our participants underwent baseline clinical examinations and MRI scans between 2014 and 2022 (mean age = 68.1, SD=5.7 years, 67% female, mean MMSE score = 29.2, SD=1.3, CDR global score ≤0.5) with annual follow‐up over 4.9 years (SD=2.5 years) and successfully completed the baseline phone assessment in 2022‐2023.

The results will be presented at the conference.

**Conclusion:**

Automated speech‐based cognitive testing may assist in low‐threshold identification of older adults at higher risk for clinical progression to AD which in turn may assist in allocation of clinical resources. In future analyses, we will further investigate associations of cognitive performance and speech features with structural or functional connectivity changes in the brain using DTI or functional MRI data.

